# Efficacy of a virtual nursing simulation-based education to provide psychological support for patients affected by infectious disease disasters: a randomized controlled trial

**DOI:** 10.1186/s12912-024-01901-4

**Published:** 2024-04-07

**Authors:** Eunjung Ko, Yun-Jung Choi

**Affiliations:** Chung-Ang University, Red Cross College of Nursing, Seoul, South Korea

**Keywords:** Virtual simulation, Psychological support, COVID-19, Infectious diseases disaster, Quarantine, Nursing education

## Abstract

**Background:**

Virtual simulation-based education for healthcare professionals has emerged as a strategy for dealing with infectious disease disasters, particularly when training at clinical sites is restricted due to the risk of infection and a lack of personal protective equipment. This research evaluated a virtual simulation-based education program intended to increase nurses’ perceived competence in providing psychological support to patients affected by infectious disease disasters.

**Methods:**

The efficacy of the program was evaluated via a randomized controlled trial. We recruited 104 nurses for participation in the study and allocated them randomly and evenly to an experimental group and a control group. The experimental group was given a web address through which they could access the program, whereas the control group was provided with a web address that directed them to text-based education materials. Data were then collected through an online survey of competence in addressing disaster mental health, after which the data were analyzed using the Statistical Package for the Social Sciences(version 23.0).

**Results:**

The analysis showed that the experimental group’s disaster mental health competence (F = 5.149, *p* =.026), problem solving process (t = 3.024, *p* =.003), self-leadership (t = 2.063, *p* =.042), learning self-efficacy (t = 3.450, *p* =.001), and transfer motivation (t = 2.095, *p* =.039) significantly statistically differed from those of the control group.

**Conclusions:**

A virtual nursing simulation-based education program for psychological support can overcome limitations of time and space. The program would also be an effective learning resource during infectious disease outbreaks.

**Clinical trial registration:**

This Korean clinical trial was retrospectively registered (21/11/2023) in the Clinical Research Information Service (https://cris.nih.go.kr) with trial registration number KCT0008965.

## Background

The last two decades have confronted the world with a variety of infectious diseases, such as severe acute respiratory syndrome, which first occurred in Asia in 2003 before spreading worldwide, including Korea, in only a few months. Since then, infectious disease outbreaks began to be recognized as severe disasters. Other examples include the 2009 H1N1 influenza outbreak, which caused more than 10,000 deaths worldwide and 140 deaths in Korea; the proliferation of the Ebola virus, which resulted in a fatality rate of more than 90% in Africa in 2014; and the outbreak of Middle East respiratory syndrome in 2015, Zika virus disease in 2016, and coronavirus disease (COVID-19) in 2019 [1]. The COVID-19 pandemic, in particular, has caused infections among approximately 64 million people and the deaths of 1.5 million individuals as of December 2020 [2].

Direct victims of infectious disease disasters, infected patients, and quarantined individuals suffer from a fear of stigma or social blame and guilt, but even people who are unexposed to sources of infection experience psychological distress from anxiety and fear of disease or possible death [3]. They also blame infected people and harbor hatred toward them [3]. This assertion is supported by an examination of web search behaviors and infodemic attitudes toward COVID-19, which identified superficial and racist attitudes [4]. Additionally, in research using a health stigma and discrimination framework related to communicable diseases, the authors found that people exhibit negative stereotypes, biases, and discriminatory conduct toward infected groups owing to fears of contagion, concerns about potential harm, and perceptions that individuals violate central values [5]. Stigmatized individuals experience adverse effects on their health because of both the stress induced by stigma and the decreased use of available services [5].

Severe and prolonged anxiety, fear, blame, and aggression can lead to mental health problems, including depression, anxiety, panic attacks, somatic symptoms, post-traumatic stress disorder, psychosis, and even suicide and life-threatening behaviors [6]. Therefore, recovery from the psychological trauma caused by a disaster should be regarded as equally necessary as physical recovery, with emphasis placed on psychological support activities that prevent the deterioration of mental health [7].

Disasters pose a significant threat to mental health support systems, wherein the lack of healthcare professionals or psychologists trained to address these conditions exacerbates the psychological distress and psychopathological risk experienced by society [8]. When training at clinical sites is restricted due to infection risks and a lack of personal protective equipment (PPE), an emerging solution is virtual simulation [9].

A virtual simulation is a simulation modality developed on the basis of video or graphic recordings featuring virtual patients and delivered via either a static or mobile device. It replicates real-world clinical situations and affords learners an interactive experience [10]. Virtual simulation-based education provides an immersive clinical environment, as virtual patients respond to a learner’s assessments and interventions [11, 12]. It enables two-way communication, and allows medical professionals to practice making clinical decisions [10]. Virtual patients are equipped with voice, intonation, and expressions that reinforce the educational narrative within the virtual environment, thereby enhancing the effectiveness of the learning experience [13]. One of the primary advantages of virtual simulation-based education is its provision of a safe and non-threatening environment in which learners can practice. It also offers flexible and reproducible learning experiences, thus catering to the diverse needs of learners [14].

Self-assessment is the most commonly used competence evaluation tool, as it is cost-effective and helps nurses improve their practice by identifying their strengths and weaknesses for development [15]. Self-assessed competence is also related to the quality of patient care because nurses promote continuous learning by determining educational needs through such evaluations [16]. The competence perceived by a nurse is inherently subjective given its self-reported nature and poses a challenge in establishing a direct correlation with the actual care of patients [17, 18]. However, studies have indicated that increased levels of self-perceived competence are associated with a significant increase in core competencies related to patient care and frequent use of clinical skills [19, 20]. Perceived competence likewise influences the job satisfaction and organizational citizenship behavior of nurses and is significantly related to absenteeism, one of the deterrents to the delivery of quality care [21, 22].

Competence refers to the possession of qualifications and abilities to satisfy professional standards, as well as the capability to perform tasks and duties in a suitable and effective manner [23]. Competencies for disaster mental health are crucial for enhancing disaster response capabilities. These competencies encompass a range of skills, knowledge, and attitudes necessary for mental health professionals to effectively support individuals and communities affected by disasters [24]. Such competencies and how they are affected by simulation-based training have been explored in some studies, which reported a significant increase in competence after exposure to the aforementioned education [25, 26].

The simulation education defined in mock training designs based on real situations provides opportunities to exercise problem-solving through various strategies. Problem-solving process is considered key competency through which learners are expected to enhance their relevant knowledge and clinical performance abilities [27]. In particular, problem-solving processes for identifying and assessing problems and finding solutions are psychological strategies that help people cope and recover after a disaster [28]. A scoping review on the effect of simulation-based education on the problem-solving process indicated that out of 32 studies reviewed, 21 demonstrated statistically significant improvement in people’s ability to resolve problems [29].

Simulation training can also address self-leadership, which is an essential self-learning quality that aids individuals in staying motivated and focused on their learning goals. It is also required as a basic qualification of professional nurses, who must be able to take initiative and make responsible decisions [30, 31]. Previous studies have reported statistically significant improvements in self-leadership following simulation training [32, 33].

Another aspect that benefits from simulation-driven education is learning self-efficacy, which plays a crucial role in predicting learners’ levels of engagement and academic success in online education. It reflects learners’ confidence in their ability to manage their own learning process. It is a significant predictor of both learners’ participation levels and their academic achievements in online education settings [34, 35]. Several studies have demonstrated virtual simulation- or online education-induced significant improvements in learning self-efficacy [36, 37]. Finally, virtual simulation-based education can also improve the motivation to transfer new knowledge and skills learned through education to clinical practice [38]. This motivation is considered an essential measure of effective learning for nurses working in the clinical field [38]. A previous study reported that psychiatric nursing simulation training combined with post-course debriefing significantly increases participants’ level of motivation to transfer [38].

On the basis of the discussion above, this study evaluated a virtual nursing simulation-based education program on disaster psychology designed to provide psychological support to patients affected by infectious disease disasters.

## Methods

### Study design

This study conducted a randomized controlled trial (RCT) to test the virtual nursing simulation-based education program of interest. The RCT protocol used was based on CONSORT guidelines.

### Participants

**Fig. 1:**
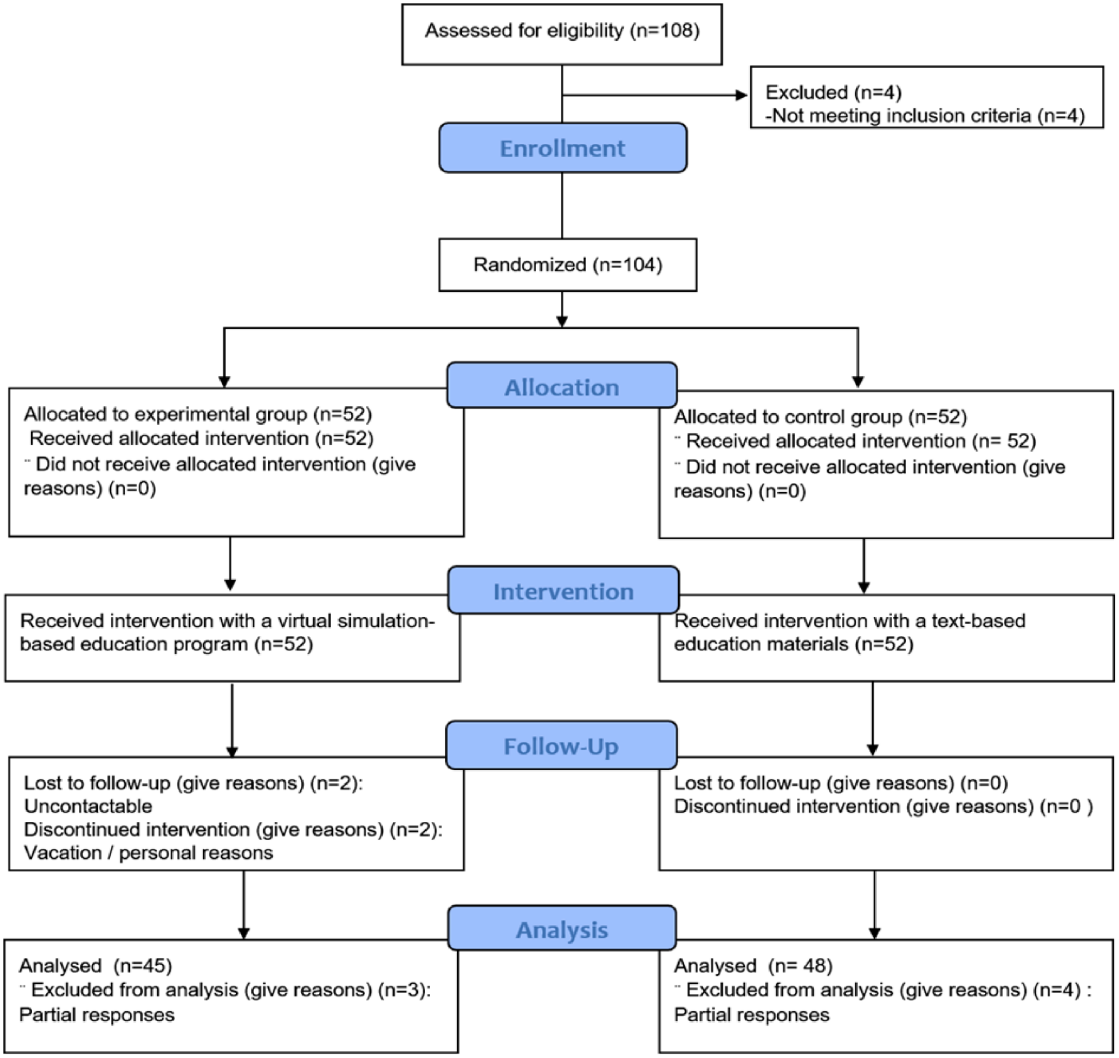
Flowchart of the randomized controlled trial

We recruited nurses working at general hospitals in South Korea. With permission from the nurse managers of these hospitals, a participation notice was posted on the institutions’ internet bulletin boards for nurses for a week. The two-sided test criterion, with a significance level (α) of 0.05, a power (1-β) of 0.80, and a medium effect size of 0.6, dictates that the minimum number of participants per group be 90. The effect size was based on a virtual simulation intervention study conducted by Kim and Choi [36]. Taking the dropout rate into consideration, we recruited 104 nurses, who were assigned to an experimental group and a control group using the random sampling functionality of the Statistical Package for the Social Sciences (SPSS version 23.0). Out of the initial sample, 11 participants were excluded because they were on vacation, could not be contacted, or provided incomplete responses during data collection (Fig. 1).

### The virtual nursing simulation-based education program

**Fig. 2:**
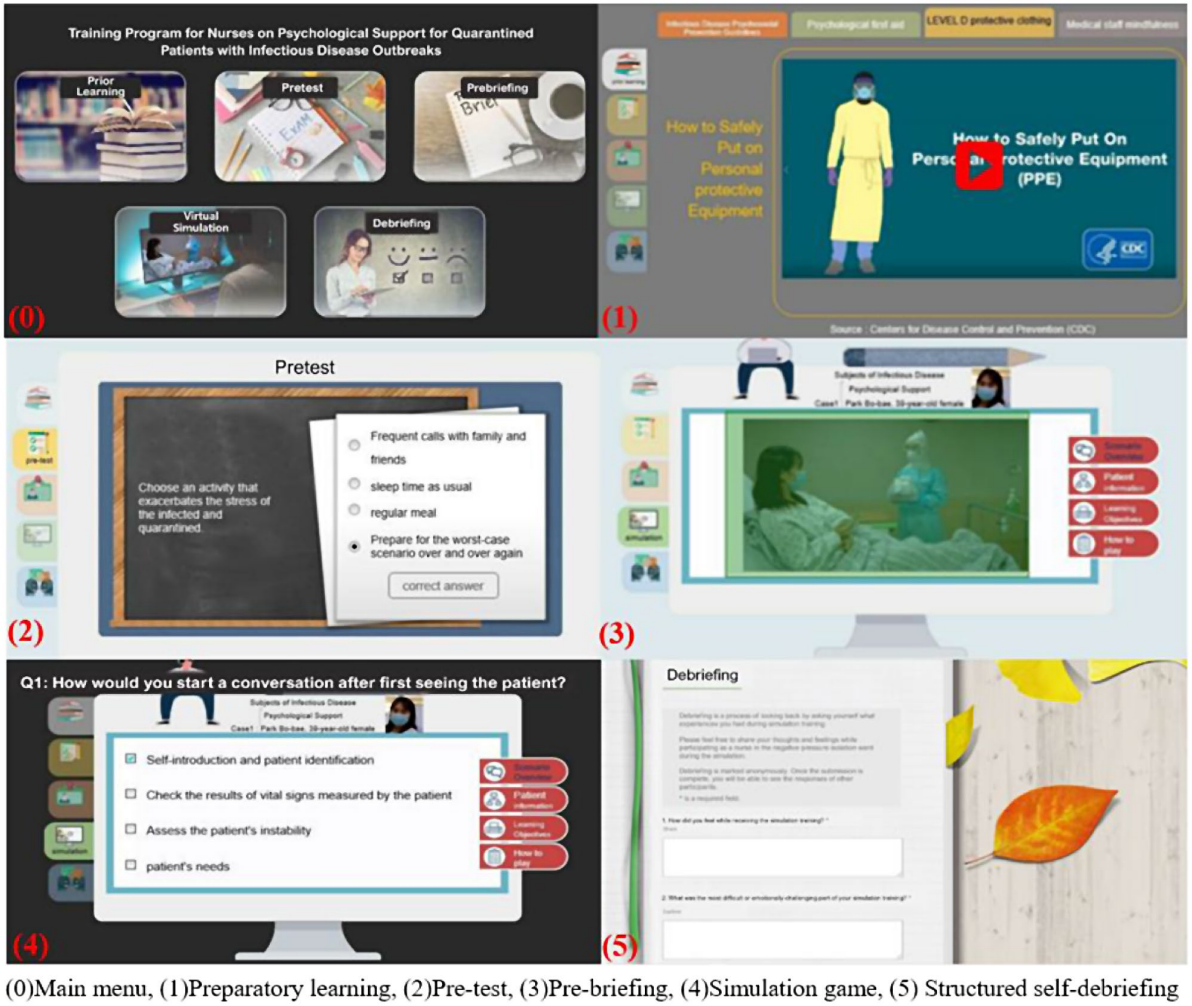
The evaluated virtual nursing simulation-based education program (examples are our own work)

This study probed into the virtual nursing simulation-based education program developed by Ko [39]. The program is implemented using an e-learning development platform, Articulate Storyline, whose operating environment is compatible with all web browsers (Internet Explorer, Microsoft Edge, Firefox, Google Chrome, etc.). It is a mobile-friendly application that can run in devices with Android and iOS operating systems. When an individual uses their smartphone or personal computer to access the server via the web address corresponding to the education program, the content functions execute. Ko’s [39] program involves five stages of learning completed in 100 min: (1) preparatory learning (30 min), (2) pre-test (5 min), (3) pre-briefing (5 min), (4) simulation game (30 min), and (5) structured self-debriefing (30 min) (Fig. 2).

Preparatory learning comes with lecture materials on guidelines for providing psychological support to victims of infectious disease disasters, administering psychological first aid, donning and doffing PPE, and exercising mindfulness through videos and pictures. In the pretest stage, a learner answers five questions and can immediately check the correct responses, which come with detailed explanations. In the prebriefing stage, an overview of a nursing simulation scenario, patient information, learning objectives, and instructions on using the virtual simulation are provided. During the simulation game, a video of the simulation is presented. It starts with a 39-year-old female, a standardized patient who is age- and gender-matched to the scenario, confirmed to have contracted COVID-19 and transferred to a negative pressure isolation room. The patient presents with extreme anxiety and feeling of tightness in her chest. During the game, learners are expected to complete 12 quizzes. In the debriefing stage, a summary of the simulation quiz results and self-debriefing questions are provided, and the comments made by learners are saved in the Naver cloud platform.

### Measurements

#### Disaster mental health competence

Disaster mental health competence was measured using the perceived competence scale for disaster mental health workforce (PCS-DMHW), which was developed by Yoon and Choi [40]. This tool consists of 24 questions related to knowledge (6 questions), attitudes (9 questions), and skills (9 questions). Each item is rated using a five-point Likert scale (0 = strongly disagree, 4 = strongly agree), and the responses are summed. The higher the score, the greater the perception of competence in a relevant area [40]. The Cronbach’s α values of the PCS-DMHW were 0.95 and 0.94 at the time of tool development and the present study, respectively.

#### Problem solving process

Problem solving process was determined using a tool modified and supplemented by Park and Woo [41] on the grounds of the problem solving process and behavior survey developed by Lee [42]. This tool is composed of 25 questions on five factors, namely, problem discovery, problem definition, problem solution design, problem solution execution, and problem solving review [41]. The reliability of the tool was 0.89 at the time of development [41], but the Cronbach’s α found in the current research was 0.94.

#### Self-leadership

Self-leadership was measured using a tool developed by Manz [43] and modified by Kim [44]. The tool consists of 18 questions distributed over six factors (three questions each): self-defense, rehearsal, goal setting, self-compensation, self-expense edition, and constructive thinking. The reliability of the tool at the time of development and the present research was (Cronbach’s α) 0.87 and 0.82, respectively.

#### Learning self-efficacy

To ascertain learning self-efficacy, we used the tool developed by Ayres [45] and translated by Park and Kweon [38]. This tool consists of 10 questions, and it had a reliability (Cronbach’s ⍺) of 0.94 and 0.93 at the time of development and the current study, respectively.

### Motivation to transfer

We used Ayres’s [45] motivation to transfer scale, which was translated by Park and Kweon [38]. Its reliability at the time of development and the present research was (Cronbach’s ⍺) 0.80 and 0.93, respectively.

### Data collection

The experimental and control groups were administered a pretest through an online survey. The web address through which the evaluated virtual simulation-based education program could be accessed was provided to the experimental group, whereas text-based education materials on psychological support for victims of infectious disease disasters were given to the control group. The groups were simultaneously sent the program’s instruction manual, and their inquiries were answered through chat. After the interventions, each participant was administered a posttest through another online survey.

### Data analysis

The collected data were analyzed using SPSS version 23.0. The homogeneity test for general characteristics between the experimental and control groups was analyzed using a t-test, a chi-square test, and Fisher’s exact test. The normality of the dependent variables was analyzed using the Kolmogorov-Smirnov test. Changes in the dependent variables between the pretest and posttest were analyzed using a paired t-test. Differences in the dependent variables before and after the groups’ use of the interventions were examined via a t-test and ANCOVA.

### Ethical considerations

We completed education in bioethics law prior to the research and obtained approval of the research proposal and questionnaire from the Institutional Review Board of the affiliated university (IRB approval number 1041078-202003-HRSB-070-01CC). A signed consent form was also obtained from each participant after the purpose and methods of the research, the confidentiality of personal information, and the voluntary nature of participation or their right to withdraw from the study were explained to them. All collected data were kept in a lockable cabinet, and electronic data were encrypted and stored. These data are to be discarded after three years.

## Results

A total of 93 participants (45 in the experimental group and 48 in the control group) were left after the exclusion of unsuitable respondents. of the between-group comparisons of the subjects indicated no significant differences between them (5% significance level) in terms of general characteristics, such as gender, age, work unit, and clinical experience (Table 1).

**Table 1:** Between-group comparison

Variable	Category	Exp.(n = 45)	Cont.(n = 48)	$\text{t}/{x}^{2}$	*p*
		Mean ± SD/ n(%)	Mean ± SD/ n(%)		
Gender	Female	44 (97.8)	48 (100.0)	1.078	0.484*
	Male	1 (2.2)	0 (0.0)		
Age (years)		32.67 ± 6.36	35.29 ± 6.92	-1.901	0.061
Work unit	General ward	12 (26.7)	7 (14.6)	8.345	0.138
	Outpatient	6 (13.3)	11 (22.9)		
	ICU	14 (31.1)	10 (20.8)		
	ER	0 (0.0)	4 (8.3)		
	Special units (OR, dialysis, endoscopy, ambulatory treatment)	7(15.6)	11(22.9)		
	Others	6 (13.3)	5(10.4)		
Clinical experience (years)		9.45 ± 6.25	11.67 ± 6.75	-1.640	0.105

*Abbreviations* Exp.=experimental group, Cont.=control group, SD = standard deviation, ICU = intensive care unit, ER = emergency room, OR = operating room

*Fisher’s exact test

The score of the experimental group on disaster mental health competence increased from 48.13 in the pretest to 70.51 in the posttest (+ 22.38), whereas that of the control group increased from 53.33 in the pretest to 68.38 in the posttest (+ 15.04). These findings reflect a statistically significant difference in competence between the groups (F = 5.149, *p* =.026). The scores of the experimental and control groups on problem solving process increased from 73.07 in the pretest to 88.24 in the posttest (+ 15.18) and from 75.75 in the pretest to 83.77 in the posttest (+ 8.02), respectively. As with the competence findings, these point to a significant difference between the groups in terms of the ability to resolve problems (t = 3.024, *p* =.003) (Table 2).

**Table 2:** Effects of virtual simulation-based education (*N* = 93)

Variables	Group	Pre-test		Post-test		t(*p)*	Difference		F(*p)*
		Mean ± SD		Mean ± SD			Mean ± SD		
Disaster mental health competence	Exp. (*n* = 45)	48.13	± 13.90	70.51	± 9.11	-10.605 (< 0.001)	22.38	± 14.16	5.149 (0.026*)
	Cont. (*n* = 48)	53.33	± 10.58	68.38	± 9.52	-12.915 (< 0.001)	15.04	± 8.07	
Problem solving process	Exp. (*n* = 45)	73.07	± 13.66	88.24	± 12.39	-9.564 (< 0.001)	15.18	± 10.65	3.024 (0.003)
	Cont. (*n* = 48)	75.75	± 14.91	83.77	± 12.78	-4.601 (< 0.001)	8.02	± 12.08	
Self-leadership	Exp. (*n* = 45)	54.87	± 6.83	59.58	± 6.99	-6.381 (< 0.001)	4.71	± 4.95	2.063 (0.042)
	Cont. (*n* = 48)	57.48	± 6.75	60.10	± 6.53	-3.792 (< 0.001)	2.63	± 4.80	
Learning self-efficacy	Exp. (*n* = 45)	55.40	± 6.27	58.84	± 6.79	-5.607 (< 0.001)	3.44	± 4.12	3.450 (0.001)
	Cont. (*n* = 48)	56.81	± 6.55	57.13	± 6.81	− 0.471 (0.640)	0.31	± 4.60	
Motivation to transfer	Exp. (*n* = 45)	49.31	± 8.92	54.29	± 7.72	-4.528 (< 0.001)	4.98	± 7.37	2.095 (0.039)
	Cont. (*n* = 48)	50.50	± 7.26	51.85	± 8.33	-1.026 (0.310)	1.35	± 9.14	

*ANCOVA conducted with the covariate of pretest value, Exp.=experimental group, Cont.=control group

The score of the experimental group on self-leadership increased from 54.87 in the pretest to 59.58 in the posttest (+ 4.71), and that of the control group increased from 57.48 in the pretest to 60.10 in the posttest (+ 2.63). These results denote a statistically significant difference in this ability between the groups (t = 2.063, *p* =.042). The scores of the experimental and control participants on learning self-rose from 55.40 in the pretest to 58.84 in the posttest (+ 3.44) and from 56.81 in the pretest to 57.13 in the posttest (+ 0.31), respectively. Again, a statistically significant difference was found between the groups (t = 3.450, *p* =.001). Their scores on motivation to transfer rose from 49.31 in the pretest to 54.29 in the posttest (+ 4.98) (experimental group) and the score increased from 50.50 in the pretest to 51.85 in the posttest (+ 1.35) (control group), pointing to a significant difference between the groups (t = 2.095, *p* =.039).

## Discussion

As previously stated, this research was evaluated a virtual nursing simulation-based education program designed to provide psychological support to patients affected by infectious disease disasters. The results showed statistically significant increases in the experimental group’s pretest and posttest scores on disaster mental health competence, problem solving process, self-leadership, learning self-efficacy, and motivation to transfer.

The experimental group achieved more statistically significant improvements in disaster mental health competence than did the control group. This finding is similar to the statistically significant increase in the average disaster mental health competence shown by providers of disaster mental health services providers and non-expert groups after PFA training involving lecture and practice [46]. It is also consistent with the significant increase in the scores of school counselors on disaster mental health competence after a lecture and simulation on PFA [25]. In their study on disaster relief workers, Kang and Choi [26] measured the participants’ performance competence in PFA after the delivery of a lecture and simulation-based education using a standardized patient. The authors found a significant increase in PFA performance competence, consistent with the present research. Since there are currently no other virtual simulation-based education programs for disaster psychological support available, we compared the effectiveness of various PFA training methods with the program assessed in the present work.

In the current research, the posttest scores of the experimental group on problem solving process significantly increased, similar to the results of Kim et al.’s study on virtual simulation- and blended simulation-based education on asthmatic child nursing [47]. Both the control and experimental groups (virtual simulation only and blended simulation featuring high-fidelity and virtual simulations, respectively) showed an increase in their problem solving process scores. These results and those derived in the present work are similar because reading and pretest phases were incorporated into the design of the previous study. Given that researchers have used commercial virtual simulations featuring avatars rather than standardized patient videos available through English-based platforms, user experiences may differ, thus requiring a qualitative analysis to identify differences. However, Kim et al. [47] did not implement a debriefing after the virtual simulation program, rendering comparison impossible. Another research reported that a multimodality simulation education that combines such methods as virtual simulation, the use of mannequins, and part-task training increase increased the scores of hospital nurses’ on problem solving process [48].

In the present work, the experimental group’s self-leadership scores increased after they used the program, and these scores were higher [49, 50]. This difference can be explained by the fact that our respondents voluntarily participated in our research given their interest in self-learning programs for disaster psychological support; even in the comparison studies, participants with stronger interest in leadership education typically exhibited heightened degrees of self-leadership [51]. The increase in self-leadership scores in the current research is consistent with a previous study involving a two-hour simulation education about PPE donning and doffing, medication administration, and medical specimen treatment in a scenario of patients suspected of having infectious diseases [32]. Another research showed that simulation education on high-risk pregnancy enhances nursing students’ problem-solving processes and self-leadership [52].

Learning self-efficacy is a key variable that enables the prediction of learners’ degrees of participation in online education and the prediction of their academic achievements, as it points to the ability to manage their learning processes [34, 53]. The results of the current research in this regard are consistent with those of a study on the online practice of basic nursing skills, which increased participants’ learning self-efficacy [54]. The researchers included an online quiz about basic nursing skills and feedback sections for learners’ self-evaluations of their performance as avenues through which to encourage autonomy in learning. A similar approach was used in the present study, which involved both a pretest for self-evaluation, direct feedback on the virtual simulation, and a self-debriefing session, enabling the participants to reflect on their simulation experiences while reviewing other participants’ answers during self-debriefing. These functions of the evaluated program were expected to factor importantly in the significant increase in the participants’ learning self-efficacy scores.

Many studies on practice education have examined participants’ motivations to transfer knowledge and skills alongside their learning self-efficacies. In the current research, the motivation to transfer scores of the experimental increased, and the difference between the two groups was statistically meaningful. This result is consistent with the findings of Park and Kweon on the simulation education about psychiatric nursing, during which post-course debriefing increased the participants’ average scores on motivation to transfer and learning self-efficacy [38]. Conversely, Kang and Kim found that a six-week simulation program for alcoholic patient care did not generate a significant increase in the participants’ motivation to transfer and learning self-efficacy scores [55]. This finding was attributed to the unfamiliarity of the local community scenario used in the research to the participants, who were in their senior year of nursing school [55]. This limitation was overcome in the current research by administering a qualitative survey of nurses’ actual demand for education on psychological support for infectious disease patients. That is, the survey presented scenarios that the participants needed.

As with other studies, the present research was encumbered by several limitations. First, the self-assessment measures used in this study may be unreliable, because they are based on individuals’ subjective perceptions and interpretations of their abilities. There is also the possibility of respondent fatigue given that the participants were compelled to answer numerous questions. Future studies should incorporate both subjective and objective measures into data collection and consider as concise an evaluation method as possible to prevent respondent fatigue. Second, this study did not establish a direct link between the obtained results and actual changes in practice or improvements in patient outcomes. We propose a follow-up study to investigate the impact of the education program examined in this study on either the mental health of patients or the quality of patient care. Third, simulation-based education tends to be accompanied with more guidance than text-based program because the former has diverse components, including quiz games, and participants are predisposed to allocate more time to simulation-based education. These may potentially influence the results. In the future, we propose to conduct research by modifying education under the same time and guided condition.

## Conclusion

This study proposed that a well-designed virtual nursing simulation-based education program can be an effective modality with which to satisfy the educational needs of nurses in the context of infectious disease outbreaks. Such programs can be easily used by nurses anywhere and anytime before they are deployed to provide psychological support to patients with infectious diseases. They are also expected to contribute to enhancing competence in addressing disaster mental health and improving the quality of care of patients afflicted with infectious diseases.

## Data Availability

The datasets used and/or analyzed in this study are available from the corresponding author upon reasonable request.

## Abbreviations

COVID-19: Coronavirus disease 2019
RCT: Randomized controlled trial
PPE: Personal protective equipment
SPSS: Statistical Package for the Social Sciences
ANCOVA: Analysis of covariance
PFA: Psychological first aid
